# Estimated Population Access to Acute Stroke and Telestroke Centers in the US, 2019

**DOI:** 10.1001/jamanetworkopen.2021.45824

**Published:** 2022-02-09

**Authors:** Kori S. Zachrison, Rebecca E. Cash, Opeolu Adeoye, Krislyn M. Boggs, Lee H. Schwamm, Ateev Mehrotra, Carlos A. Camargo

**Affiliations:** 1 Department of Emergency Medicine, Massachusetts General Hospital, Boston; 2 Department of Emergency Medicine, Harvard Medical School, Boston, Massachusetts; 3 Department of Emergency Medicine, Washington University, St Louis, Missouri; 4 Department of Neurology, Massachusetts General Hospital, Boston; 5 Department of Neurology, Harvard Medical School, Boston, Massachusetts; 6 Department of Health Care Policy, Harvard Medical School, Boston, Massachusetts

## Abstract

This cross-sectional study assesses US population access to emergency departments with acute stroke capabilities and telestroke capacity in 2019.

## Introduction

Ongoing efforts to improve stroke systems of care aim to provide all patients with sufficient access to time-sensitive reperfusion interventions.^1^ Emergency departments (EDs) vary in their capabilities and resources, typically reflected by their stroke center status: (1) acute stroke-ready hospitals care for patients with stroke in the acute phase but patients may require a subsequent transfer, (2) primary stroke centers have capabilities beyond the acute phase, and (3) thrombectomy-capable and comprehensive stroke centers have the most specialized personnel and resources.^2^ Emergency departments with telestroke capabilities can also quickly access the necessary expertise to treat stroke.

Previous work has demonstrated variation in US population access to stroke center hospitals, with nearly 20% of Americans lacking timely access to alteplase-capable hospitals in 2011.^3^ In the interim, there has been a substantial investment in both increasing the number of stroke centers and expanding the use of telestroke services. To provide an update on population-level access to stroke care, we (1) estimated the proportion of the US population with access to an ED with acute stroke capabilities (ie, in a confirmed stroke center or with telestroke capacity) and (2) assessed the specific contribution of telestroke services to US population access.

## Methods

This cross-sectional study was approved by the Mass General Brigham Institutional Review Board. Informed consent was not required because the study did not involve individual patient data. The study followed the Strengthening the Reporting of Observational Studies in Epidemiology (STROBE) reporting guideline.

We used the 2019 National Emergency Department Inventory (NEDI)-USA to identify all US EDs open in 2019. We categorized each ED as having telestroke capacity (yes or no, self-reported based on the NEDI survey; 14% of EDs with missing data were classified as no)^4^ and whether it was part of a hospital stroke center (including acute stroke-ready hospitals, classified as yes or no).

Our unit of analysis was at the census block group based on 2020 US Census data, with each population-weighted centroid calculated using ArcGIS Desktop 10.7 (Esri). We determined whether each census block had access to an ED with acute stroke expertise within a transport time of 60 minutes. Prehospital transport times were derived from the 2019 National Emergency Medical Services Information System, including emergency medical services dispatch, response, scene times, and ground transport driving times, stratified by census division and urbanicity. No statistical testing was performed; further details are available in the eMethods in the Supplement.

## Results

In 2019, there were 5587 EDs open in the US. Of these, 2563 (46%) were in stroke centers and 2505 (45%) were categorized as having telestroke services in the ED. Our results indicate that 1101 of 3024 EDs (36%) not in a stroke center had telestroke capacity compared with 432 of 691 EDs (63%) in acute stroke-ready hospitals and 872 of 1505 EDs (58%) in primary stroke centers.

The proportion of the US population within 60 minutes of an ED in a stroke center was 91% (Figure; Table). A similar proportion of the population was within 60 minutes of a telestroke ED (90%). Finally, 96% of the population had access within 60 minutes to an ED with any acute stroke capabilities (eg, ED in a stroke center or with telestroke). Access varied by region, from 91% in the Mountain West to 99% in the Mid-Atlantic.

**Figure.**
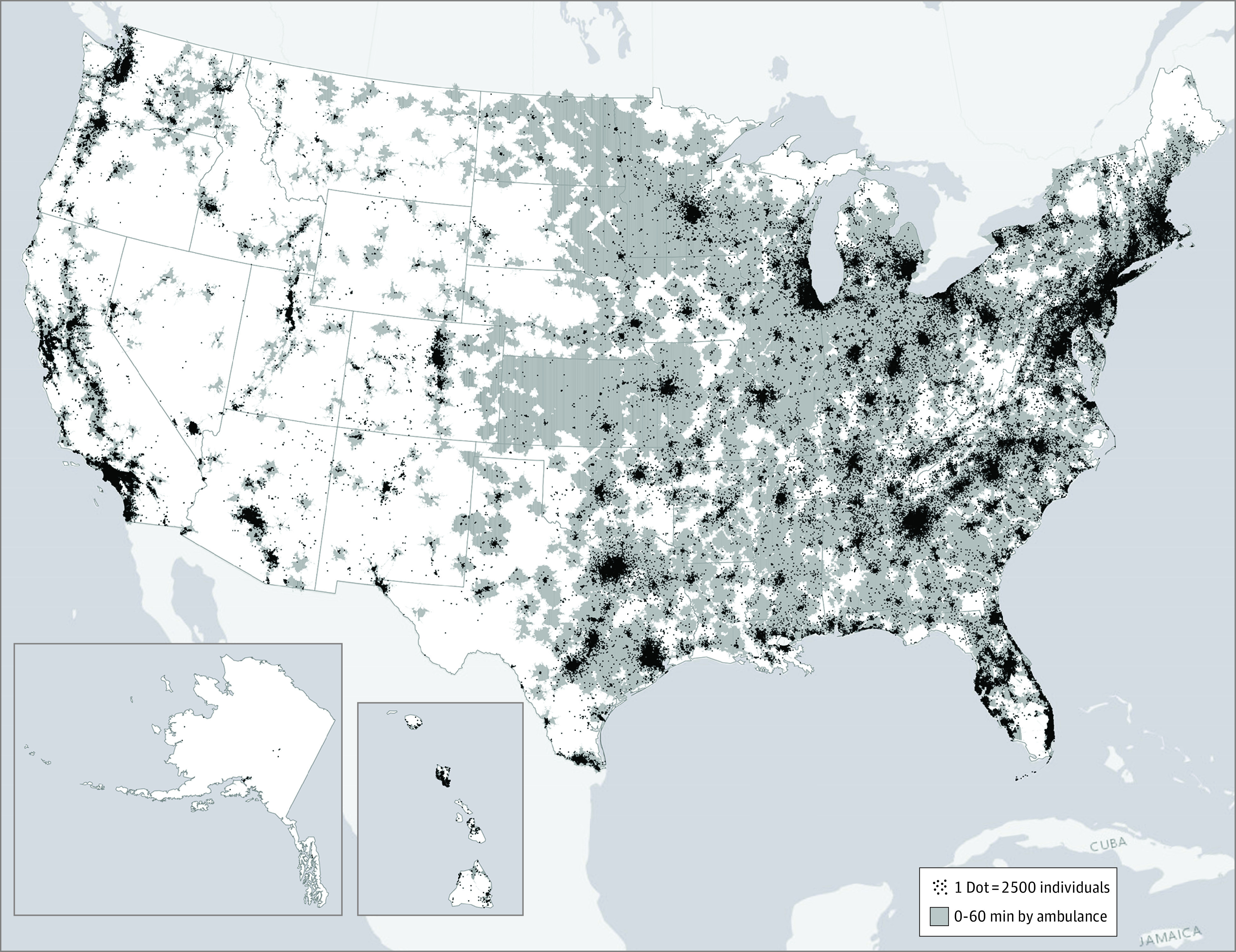
US Population Access to Emergency Departments With Acute Stroke Expertise Acute stroke expertise is defined as follows: (1) confirmation of a hospital as an acute stroke-ready hospital, a primary stroke center, a thrombectomy-capable stroke center, or a comprehensive stroke center or (2) receipt of emergency department telestroke services.

**Table.** US Population Access to Stroke Expertise

Population access^a^	Individuals, No. (%) (N = 331 449 281)
Telestroke capacity or within any stroke center	318 258 272 (96)
Telestroke ED^b^	299 585 920 (90)
Stroke center of any type^c^	302 909 696 (91)
Comprehensive or thrombectomy-capable stroke center	211 918 960 (64)
Comprehensive, thrombectomy-capable, or primary stroke center	290 007 904 (87)

^a^ Population access was defined as a transport time of 60 minutes or less to an emergency department (ED) with stroke expertise.

^b^ Telestroke ED only considers telestroke capacity; all EDs with telestroke, regardless of stroke center status, are included here.

^c^ Stroke center includes acute stroke-ready hospital, primary stroke center, thrombectomy-capable stroke center, or comprehensive stroke center.

Of the 13 191 024 people (4% of the total population) without 60-minute access, 7 905 276 (60%) were within 60 minutes of a nontelestroke, nonstroke center ED.

## Discussion

Overall, we observed a substantial increase in population access to acute stroke care in this cross-sectional study relative to previous reports using alternative methods.^3^ This increase likely reflects the extensive and ongoing work to improve stroke systems of care, including greater stroke center accreditation and expansion of telestroke capacity.

A major study limitation was self-reported telestroke capacity that was not confirmed by our study team. Despite improvements in stroke systems of care, many Americans still lack timely access to acute stroke intervention. Although the smaller, critical access hospitals serving patients in rural areas are the most likely to benefit from telestroke services,^5^ they are currently the least likely to have them.^4,6^ Addressing this care gap and other disparities in access will be critical to improving equitable access to acute stroke care for all Americans.

## Notes

Supplement.
eMethods.

eReferences
