# Supplementary material for: Estimated Population Access to Acute Stroke and Telestroke Centers in the US, 2019
Source: JAMA Netw Open. 2022 Feb 9;5(2):e2145824. doi: 10.1001/jamanetworkopen.2021.45824 (PMC8829668; doi:10.1001/jamanetworkopen.2021.45824)
Supplement: Supplement. — eMethods. eReferences [file jamanetwopen-e2145824-s001.pdf]

Supplementary Online Content

Zachrison KS, Cash RE, Adeoye O, et al. Estimated population access to acute stroke and telestroke centers in the US, 2019. *JAMA Netw Open*. 2022;5(2):e2145824. doi:10.1001/jamanetworkopen.2021.45824

eMethods.
eReferences

This supplementary material has been provided by the authors to give readers additional information about their work.

© 2022 Zachrison KS et al. *JAMA Network Open*.

eMethods

This report follows the STROBE reporting guideline for cross-sectional studies.

Additional Details of Emergency Department (ED) Sample and Identification of EDs' Telestroke Capability and Stroke Center Status

We used the 2019 National ED Inventory (NEDI)-USA to identify all US EDs that were open in 2019. EDs were included if they operated 24 hours per day, 7 days per week during the year, including hospital based EDs, satellite freestanding EDs, and autonomous freestanding EDs. NEDI-USA is an annual survey of all US EDs, including the increasing number of free-standing EDs.[1] Since 2016 NEDI-USA has included questions on the use of telestroke in the ED. ED directors are asked the question: "Does your ED receive telemedicine services for patient evaluation…" and if "YES, *(check all that apply)*" with "Stroke/neuro" as a listed option.[2] The overall response rate to the 2019 survey was 86%. EDs with missing responses or responses indicating the ED did not use any telehealth services were categorized as not having telestroke.

Hospitals, not the EDs per se, are certified as stroke centers. We determined whether an ED is located in a stroke center by using data from the Joint Commission (TJC), Det Norske Veritas (DNV), and Healthcare Facilities Accreditation Program (HFAP). We also identified hospitals designated by states as a stroke center by contacting representatives from the public health department or emergency medical services in each state. We reviewed minimum requirements at the state level and standardized state stroke center status to TJC classification standards (ASRH, PSC, or TSC/CSC). No EDs had missing stroke center status.

Additional Details of Travel Time Calculations and Data Analysis

We measured the median prehospital time for actual emergency medical services (EMS) stroke transports from the 2018 and 2019 National EMS Information System (NEMSIS) Version 3.4 Public Release Research dataset.[3] Prehospital time included time for EMS dispatch, response, and time on scene. Prehospital time was stratified by US Census division and urbanicity.

To assess whether a given census block had access to reperfusion treatment withing 60 minutes, we determined the maximum possible driving time from the scene to ED. Similar to prior work,[4] travel time calculations incorporated EMS dispatch, response, and scene times plus actual driving times by ground transport based on posted speed limits without traffic. For example, in suburban settings in New England, the median dispatch, response and scene times were 1.0, 6.3, and 14.9 minutes, respectively. This gives 22.2 minutes of prehospital time. We then used ArcGIS Network Analyst to determine whether there was an ED within 37.8 minutes (60-22.2) driving time of that population centroid. For each population centroid we determined whether it was within 60 minutes total ground transport time from a telestroke ED, an ED in an ASRH, a PSC, or a TSC/CSC. We calculated the total population that fell within the drive time areas for each type of ED. We also examined results stratified by Census division to evaluate for regional differences in access.

© 2022 Zachrison KS et al. *JAMA Network Open*.

eReferences

1. Sullivan AF, Richman IB, Ahn CJ, Auerbach BS, Pallin DJ, Schafermeyer RW, Clark S, Camargo CA. A Profile of US Emergency Departments in 2001. *Ann. Emerg. Med.* 2006;48:694–701.
2. Zachrison KS, Boggs KM, M Hayden E, Espinola JA, Camargo CA. A national survey of telemedicine use by US emergency departments. *J. Telemed. Telecare*. 2020;26:278–284.
3. Mann NC, Kane L, Dai M, Jacobson K. Description of the 2012 NEMSIS public-release research dataset. *Prehosp. Emerg. Care*. 2015;19:232–240.
4. Adeoye O, Albright KC, Carr BG, Wolff C, Mullen MT, Abruzzo T, Ringer A, Khatri P, Branas C, Kleindorfer D. Geographic access to acute stroke care in the United States. *Stroke*. 2014;45:3019–24.

© 2022 Zachrison KS et al. *JAMA Network Open*.
